# Dapoxetine versus glans penis injection with hyaluronic acid gel in treatment of premature ejaculation

**DOI:** 10.1080/2090598X.2023.2245598

**Published:** 2023-08-11

**Authors:** Ibrahim M. Ibrahim, Moustafa Mohamed, Mostafa Kamel, Lotfy Elbendary, Almaqtouf Mohamed, Mohamed S Elderey

**Affiliations:** a Faculty of Medicine, Department of Urology, Zagazig University, Sharkia, Egypt; b Faculty of Medicine, Department of Urology, Zagazig University, benghazi, Libya

**Keywords:** Hyaluronic Acid Gel, Dapoxetine, premature ejaculation and glans penis injection

## Abstract

**Objective:**

to compare the results of using Dapoxetine and HA (hyaluronic acid) gel injection by Five puncture technique in the treatment of premature ejaculation (PE).

**Methods:**

100 sexually active heterosexuals circumcised males with lifelong PE were included in the study. Group A patients were treated with on-demand Dapoxetine, while group B was treated with HA gel glans penis injection using a five-puncture technique. Both groups were evaluated at 1^st^,3^rd^ and 6^th^ months post-treatment using IELT.

**Results:**

There were no significant differences between both groups regarding patient demographic. Mean pretreatment IELT in groups A and B were 45.82 ± 7.44 and 46.18 ± 7.82 receptively. There was no significant difference between both groups. After treatment, both groups show significant ILET improvement during the 1^st^,3^rd^, and 6^th^ months follow-up with a *P* value < 0.001. However, when comparing the improvement of ILET in group A (Dapoxetine) and group B (HA injection), there were high significance differences in favor of group B in the 1st,3rd, and 6th-month follow-up.

**Conclusion:**

Although both treatment modalities have improved IELT and premature ejaculation, but HA injection with five punctures technique was significantly better than oral Dapoxetine with self-limited side effects.

## Introduction

Premature ejaculation (PE) is reported as a common male sexual problem and it affects nearly 20% of the sexually active male population [1,2]. Many definitions were developed to define premature ejaculation. The definitions emphasize poor self-control, lack of satisfaction, and short interval from penetration to ejaculation [3]. Lifelong PE is defined as ejaculation that occurs between 30 and 60 seconds from penetrative sexual intercourse nearly all times [4]. It is worth to mention that many theories have been developed to explain PE like psychological theory, hormonal, genetic and chronic prostatitis [1,2].

5- Hydroxytryptamine 1A (5-HT1A) hypersensitivity and peripheral penile hypersensitivity are blamed to have a role in lifelong PE [5]. Acquired PE occurs in men with previous normal sexual life [4].

Many drugs were used to treat PE like antidepressants and local anesthetics. Dapoxetine is the only licensed short-acting selective serotonin reuptake inhibitor for treating PE. It prevents serotonin transportation so it increases its level at the post-synaptic cleft and as a result it delays ejaculation [6]. But the recurrence rate of PE after stopping treatment and the systemic side effects create a need for seeking other treatment options [7].

Hyaluronic acid (HA) gel penile injection is a promising treatment for PE. It lowers the level of stimulation of the penile skin receptors. Many studies reported that HA penile injection increased the intra-vaginal latency time (IELT) about four to five times and this effect may continue for 5 years [8,9]. The most reported side effects were transient discoloration and swelling of the glans. Most of them resolved within 2 weeks [9]. Also, HA injection allows spontaneous relationship.

we aim in this study to compare the effectiveness and safety of Hyaluronic acid gel injection versus dapoxetine in treatment of premature ejaculation.

## Patient and methods

This study is a comparative randomized controlled clinical trial using Dapoxetine (group A) and HA (hyaluronic acid) gel injection by Five puncture technique [10] (group B) for PE treatment. Patients included in the study were sexually active heterosexual circumcised males with lifelong PE. PE diagnosis relied on the standards from the International Society for Sexual Medicine (ISSM), using IELT stopwatch testing. The baseline period was 4 weeks for assessment. All participants were required to have at least four sexual intercourses. Patients with chronic psychiatric, systemic disease, endocrine disorders, drug abuse, prostatitis, erectile dysfunction, acquired PE, and those who recently received treatment for PE were excluded.

**Figure 1.**
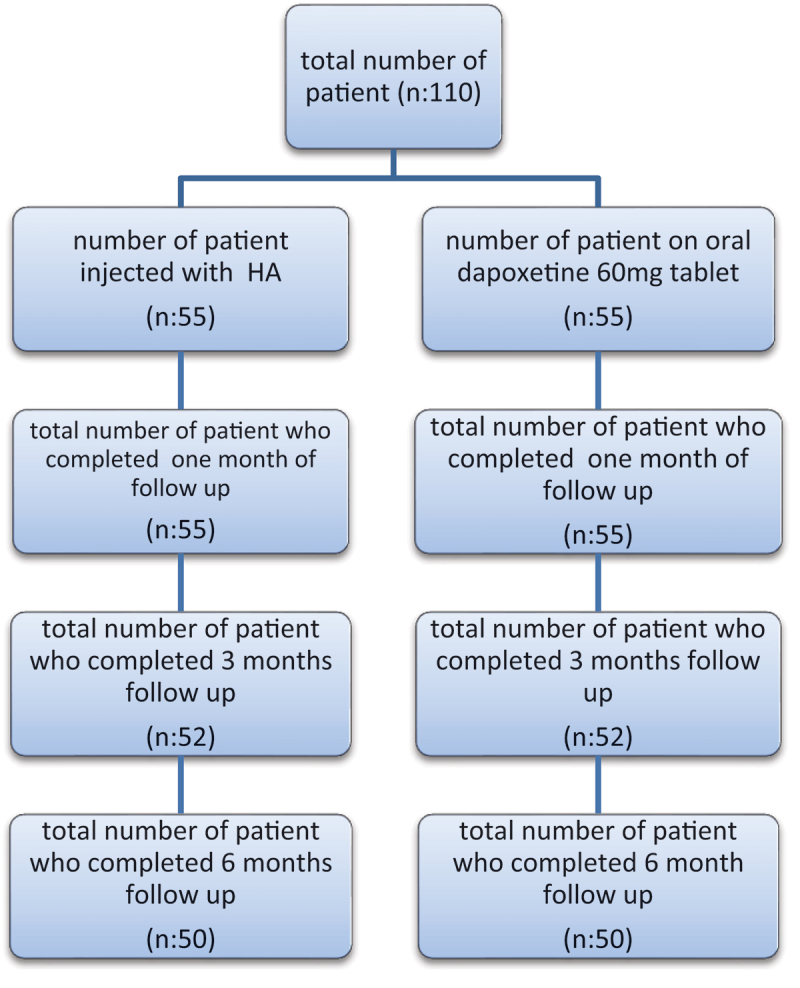
Total number of patient in each group.

The sample size was 110, with 55 patients for each group. It was calculated using open Epi assuming that difference in IELT (3.2+_ 0.13 seconds) among the group injected with HA versus (2.4+_ 2.14 seconds) among the Dapoxetine group. However, 10 patients discontinued the study (5 in both groups during follow-up) (Figure 1). Patients were randomized according to the closed-envelope method. Group A patients were treated with Dapoxetine 60 mg tablet on demand given 3 hours before the sexual episode. Group B patients were treated by injection of 2 ml hyaluronic acid (HA; STYLAGE® IPN Like TECHNOLOGY, VIVACY Laboratories, Paris, France) in the glans penis by 5 puncture technique [10].

**Figure 2.**
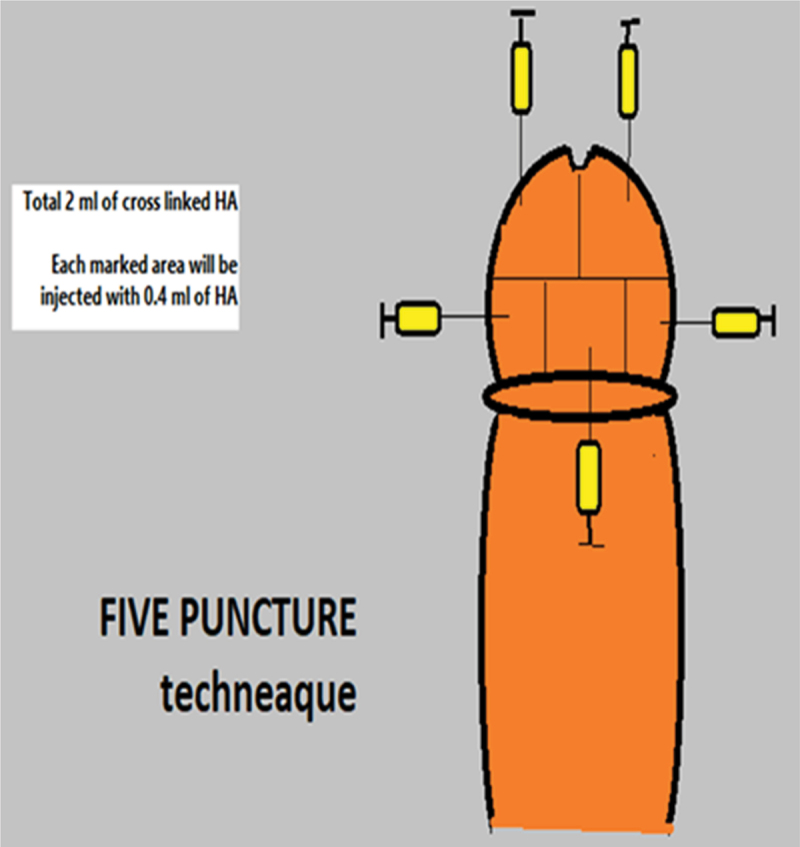
5 puncture technique Sakr a et al 2022(10).

Topical local anaesthetic (Xylocaine Jelly 2%:lidocaine 20 mg (Aspen, Sweden) was applied 30 minutes before injection using 30-gauge needle. The glans is divided by horizontal line into two halves with further division of the distal one into two halves; right and left using a vertical line. The proximal half is divided into three parts using two vertical lines. Injection of 0.4 ml of HA into the deep dermis of each part with a total dose 2 ml (Figure 2) [10].

Both groups were evaluated at 1^st^,3^rd^ and 6^th^ months post-treatment using IELT.

Statistical analysis: Data analysis was done using IBM SPSS 23.0 for Windows (SPSS Inc., Chicago, IL, USA) and NCSS 11 for Windows (NCSS LCC., Kaysville, UT, USA). Qualitative data is represented as number and percentage, and quantitative is continuously represented by mean and standard deviation. The following tests were used to test differences for significance; difference, and association of qualitative variable by Chi-square test (X^2^). Differences between quantitative independent groups by t-test or Mann-Whitney for non-parametric data. Repeated measures ANOVA for comparison of multiple means. *P* value was set at < 0.05 for significant results &<0.001 for high significant results.

## Results

### Patients’ socio-demographic data

In group A mean patients’ age and BMI were (40.73 ± 10.98) years and (25.44 ± 6.31) receptively. While in group B patient’s age and BMI were (42.31 ± 9.78) years and (25.3 ± 5.99). The mean wife age was 30.57 ± 6.78 years in group A and 32.41 ± 7.18 years in group B. Duration of marriage and frequency of intercourse in group A were 8.05 ± 5.12 years and 2.22 ± 0.93 receptively while in group B were 9.54 ± 5.1 years and 1.89 ± 0.92. There was no statistically significant difference among both studied groups regarding socio-demographic characters, both groups were matched (Table 1).

**Table 1.** Socio-demographic data.

			Group A	Group B	t-test	P
Age	Mean± SD		40.73 ± 10.98	42.31 ± 9.78	0.79	0.43
	Range		20–62	25–66		
BMI		Mean± SD	25.44 ± 6.31	25.3 ± 5.99	0.08	0.94 NS
		Range	16–36	15–36		
			Group A	Group B	MW\t-test*	
			Mean SD	Mean SD		
Wife age (years) Range			30.57 ± 6.78	32.41 ± 7.18	1.38	0.17 NS
			20–47	20–47		
Duration of marriage (years) Median (Range)			8.05 ± 5.12	9.54 ± 5.1	1.58	0.14 NS
			7 (2–19	10 (1–22)		
Frequency of intercourse (Per week) Median (Range)			2.22 0.93	1.89 0.92	1.99	0.05 NS
			2 (1–4)	2 (1–4)		

There was no statistical significant difference among both studied groups regarding socio-demographic characters, both groups were matched.

### Change in pre and post-treatment IELT

**Figure 3.**
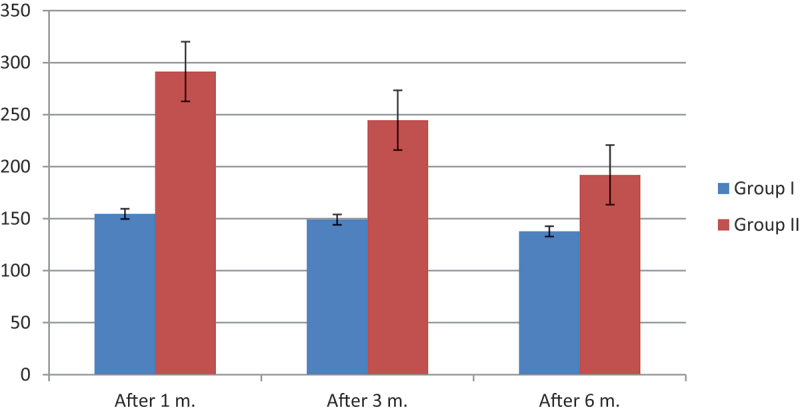
Difference in IELT among both studied groups post-treatment.

Mean pretreatment IELT in groups A and B were 45.82 ± 7.44 seconds and 46.18 ± 7.82 seconds receptively. There was no significant difference between both groups. After treatment, both groups show significant ILET improvement during the 1st,3rd, and 6th-month follow-up with a *P* value < 0.001. However, when comparing the improvement of ILET in group A (Dapoxetine) and group B (HA injection), there were high significant differences in favor of group B in the 1st,3rd, and 6th-month follow-up (Table 2)(Figure 3).

**Table 2.** Pretreatment and post treatment IELT.

IELT	Group A	Group B	t-test	P
	Mean ± SD	Mean ± SD		
Pre-	45.82 ± 7.44	46.18 ± 7.68	0.25	0.807NS NS
After 1 month	154.6 ± 32.17	291.4 ± 9.74	30.1	<.001(HS)
After 3 months	149.1 ± 2.12	244.7 ± 12.8	28.9	<.001(HS)
After 6 months	137.8 ± 21.6	192.1 ± 9.32	16.3	<.001(HS)
Significance test P value	<0.001HS HS	<0.001HS HS		

There was no significant difference between both groups regarding pretreatment IELT. After treatment both groups show significant ILET improvement during 1st,3rd and 6th month follow up with *P* value < 0.001. However when comparing improvement of ILET in group A (Dapoxetine) and group B (HA), there were high significance differences in favor of group B in 1st,3rd and 6th month follow up.

### Post-intervention complications and adverse effects

The adverse effects of oral Dapoxetine use were nausea in six cases (12%), dizziness in six cases (12%), headache in five cases (10%), dry mouth in 4fourcases (8), and diarrhea in three cases (6%) (Table 3).

**Table 3.** Adverse effect of oral dapoxetine 60 mg tablet use in group a and complication of HA injection in group B post intervention.

(Dapoxetine) Group A		
complain	Patients	
	N	%
Nausea	6	12
dizziness	6	12
Headache	5	10
Dry mouth	4	8
Diarrhea	3	6
Group B (HA injection)		
complain	Patients	
	N	%
Pain at site of injection	7	14
Bullae formation at site of injection	4	8
Ecchymosis	3	6

This table show the adverse effect with oral dapoxetine use were nausea in six cases (12%), dizziness in six cases (12%), headache in five cases (10%),dry mouth in four cases (8), and diarrhea in three cases (6%). the complication with HA injection were pain at site of injection in seven cases (14%), bullae formation at site of injection in four cases (8%), ecchymosis in three cases (6%), which resolved completely after 10 to 15 days post injection.

The complications with HA injection were pain at the site of injection in seven cases (14%), bullae formation at the site of injection in four cases (8%), and ecchymosis in three cases (6%) (Table 3)

All the previous complications resolved completely after 10 to 15 days from injection and were well tolerated by the patients.

## Discussion

Premature ejaculation (PE) is considered a common sexual problem. Some studies reported a prevalence between 2–23% [11]. Many theories were used to explain the etiology of PE like Chronic prostatitis and psychological causes such as anxiety. Also, genetic causes and hormonal factors were suggested as a cause of PE [1,2].

Saleh R et al., 2021 in a systematic review study on the treatment of PE stated that patients with PE respond differently to various treatment options. So, urologists need to be aware of different modalities which can fulfill the patients’ needs [12].

4The treatment options for PE include on-demand Dapoxetine hydrochloride, and short-acting selective serotonin reuptake inhibitor (SSRI). Also, other daily used antidepressants and on-demand topical anesthetics were used [13].

Regarding the improvement in IELT in this study, the pretreatment means IELT was 45.82 ± 7.44 and 46.18 ± 7.82 seconds in group A and group B receptively without significant difference between both groups. The mean IELT after one month was 154.6 and 291.4 seconds in group (A) and group (B), respectively. After 3 months, it was 149.1 seconds in group (A), and 244.7 seconds in group (B). It was 137.8 seconds in group (A), and 192.1 seconds in group (B) after 6 months. There was a significant difference between the follow-up periods within the group. The post-treatment IELT was highly significantly improved for both groups. However, in comparing both groups, group B with HA injection had significantly higher IELT in comparison to group A treated by Dapoxetine in all periods of follow up at 1st,3rd, and 6th month follow up.

Regarding the improvement of IELT post-HA injection, it was in agreement with Sakr A et al., 2022. They had shown significant improvement in IELT during one year of follow-up. Despite there being a drop in IELT in the 3rd, 6th, and 12th months than the 1st month of treatment, IELT was still significantly higher than pre-injection IELT [10].

Also, Abdallah H et al., 2012 showed a significant IELT increase in the 1st-month post-HA. However, it was still significantly better than baseline IELT. HA injection gave PE patients and their partners more satisfaction than local anesthetics agents and condoms due to its prolonged effect without affecting the pleasure of the other partner [14]. Abdelazeem M et al., 2019 found that HA injection significantly improved IELT within 6 months from 88.34 ± 3.14 to 192.5 ± 7.6 seconds [11].

While in patients treated by Dapoxetine, many randomized controlled studies include more than six thousand patients with PE who studied the effect of dapoxetine in the treatment of PE. Dapoxetine had shown significant improvement in baseline IELT from (0.8 min) to (2.3 min) [15]. Also, many other studies had shown that Dapoxetine improves baseline IELT [13,16]. Although Dapoxetine has favorable outcomes in the treatment of PE, the integrated analysis of clinical trials using Dapoxetine had shown about 30% of patients included in the clinical trials had discontinued it. It was either lack of satisfaction with its results or personal issues [16]. It was reported that oral Dapoxetine significantly improved baseline IELT up to three folds. However, by using Global Efficacy Question (GEQ), only 29% of treated patients showed satisfaction with treatment results. This could be explained by that the mean baseline IELT was 20 seconds. Even if it was improved by Dapoxetine by three folds, it was still not satisfactory to the patient [17]. Many studies had reported that oral Dapoxetine use did not give satisfactory results in patients with severe PE who ejaculate rapidly within seconds [18,19]

In our study, the complications with HA injection in our study were pain at the site of injection in seven cases (14%), bullae formation at the site of injection in four cases (8%), and ecchymosis in three cases (6%). However, all these complications were self-limited within 2 weeks and patients were satisfied. One of the strength points added to the HA injection group is the possibility of a spontaneous relationship. This was in agreement with Abdallah H et al., 2012 who had shown that the complications of HA injection were mild pain and bullae formation at the site of injection [14]. Abdelazeem M et al., 2019 mentioned that HA injection had no adverse effects on over 20 patients included in the study [11]. Many other studies had revealed that the side effects of HA injection were mild and self-limited like ecchymosis and injection-site discomfort. This could be explained by the nature of HA as it is a polysaccharide that is naturally found in the intercellular matrix of human dermal layers so, it doesn’t cause a foreign body reaction [20,21].

In the current study, the adverse effect of oral Dapoxetine use was nausea in six cases (12%), dizziness in six cases (12%), headache in five cases (10%), dry mouth in five cases (8), and diarrhea in three cases (6%). Although five patients in this group dropped out during 1st follow-up visit and this may be explained by intolerable side effects. Minestrone V., et al 2014 showed that Dapoxetine use had side effects like nausea (17.3%), dizziness (9.4%), headache (7.9%), diarrhea (5.9%), somnolence (3.9%), fatigue (3.9%), insomnia (3.8%) and nasopharyngitis (3.1%). Side effects were more in patients aged 65 years or older [22]. Alghobary M ., et al 2020 reported that Dapoxetine had many side effects like nausea (20%), headache (14.5%), dizziness (10.9%), diarrhea (10.9%), and insomnia (7.3%) [17].

Our limitations in this study were the need for larger sample size and longer follow up time. Also, to assess the long-term quality of life and patient satisfaction.

## Conclusions

Although both treatment modalities have improved IELT and premature ejaculation HA injection with five punctures technique was significantly better than oral Dapoxetine with lower self-limited side effects.
